# Inflammatory bowel disease—the role of cross-sectional imaging techniques in the investigation of the small bowel

**DOI:** 10.1007/s13244-014-0377-6

**Published:** 2014-12-24

**Authors:** Athanasios Athanasakos, Argyro Mazioti, Nikolaos Economopoulos, Christina Kontopoulou, Georgios Stathis, Dimitrios Filippiadis, Themistoklis Spyridopoulos, Efthymia Alexopoulou

**Affiliations:** 2nd Radiology Department, General University Hospital ATTIKON, 1, Rimini str, Chaidari-Athens, 12462 Greece

**Keywords:** Inflammatory bowel disease, Paediatric, CT, MRI, Ultrasound

## Abstract

**Abstract:**

Background: The diagnosis of inflammatory bowel disease (IBD) in children and adolescents is based on the integration of clinical, biological, endoscopic, histological and radiological data. Methods: The most important part of the diagnosis is the histology, which is acquired by endoscopy. Imaging of the small bowel has changed in recent years, but the imaging goals are primarily to determine the extent of small bowel involvement, assess complications and define candidates for surgery. Imaging techniques are divided into conventional and cross-sectional ones. Results: The spectrum of imaging findings of cross-sectional techniques is discussed, emphasising the advantages and limitations of each technique, acknowledging the specificities of the paediatric population. Cross-sectional techniques have advanced the ability to diagnose and monitor inflammatory disease of the small bowel. Conclusion: MR enterography is the technique of choice in children with known IBD, for the investigation of the small bowel and the whole GI tract. US should be the first choice examination in children with suspected IBD, while CT should be reserved for cases in which MRI is contraindicated or in acute emergency situations when US is inadequate.

***Teaching Points*:**

• *Cross-sectional imaging of the small bowel is essential in paediatric IBD*.

• *Endoscopy is unable to assess extramural disease and examine the entire small bowel*.

• *US should be the first choice examination in children with suspected IBD*.

• *MR enterography is the technique of choice in children with known IBD*.

• *There are still controversies regarding the prediction of disease activity or fibrosis*.

## Introduction

Inflammatory bowel disease (IBD) consists of a range of diseases, which include Crohn's disease (CD), ulcerative colitis (UC) and IBD unclassified (IBDU). Approximately 1.6 million Europeans are affected with CD and 2.1 million with UC, of whom 25 % of patients first present during childhood or adolescence. Recent epidemiologic studies have documented an increase in the incidence of paediatric IBD [1].

UC is characterised by continuous mucosal, colonic inflammation, while CD by skip lesions and transmural inflammation, which may affect any part of the digestive tract. IBDU (9 % of paediatric IBD) refers to cases that cannot be classified after complete clinical, radiological, endoscopic and pathological evaluation, mainly because they present with features of both diseases [2].

Environmental changes, genetic factors, intestinal microbiota alterations and immune system deregulation contribute to the initiation and progression of inflammation and consequently fibrosis, the two main components of IBD [3].

Paediatric IBD (PIBD) is characterised by different atypical phenotypes from the adult-onset disease because of a different genetic basis and age-related regulation of the inflammatory process [4]. Atypical presentation of UC includes rectal sparing in untreated patients, short duration of disease, upper gastrointestinal (GI) tract involvement, caecal patch and acute transmural disease [2]. Location and severity of CD lesions in children are more extensive and aggressive in the left colon, while terminal ileitis is the usual pattern of inflammation in adults [4]. IBD phenotypes are reported related to the clinical outcome, so accurate characterisation is of great importance. The most recently validated Paris classification includes location, severity, morphology (structuring, penetrating), patient’s age and growth delay [5].

The diagnosis of PIBD is based on the integration of clinical, biological, endoscopic, histological and radiological data, and no single study is diagnostic. According to the revised Porto criteria, the recommendations for diagnosis include esophagogastroduodenoscopy and ileocolonoscopy with random biopsies from all segments of the GI tract, while adequate imaging studies of the small bowel (SB) are recommended in all suspected cases of IBD, particularly patients with suspected CD, atypical UC and IBDU [2]. While endoscopy is the gold standard modality as it provides a definite diagnosis, it has certain limitations: invasiveness, need for sedation, inability to assess extramural disease and inadequate visualisation of SB. Capsule endoscopy is a relatively new technique, which can be applied in children for evaluation of SB, with high specificity and sensitivity in CD, comparable to MR enterography with the main drawback of low specificity in the evaluation of the jejunum [6]. Its main disadvantages consist of: inability to evaluate extramural pathology, contraindication of strictures, 15–27 % incomplete recording and 8 % retention risk. Recently, in order to avoid retention risk, a new dissolving test capsule—named the patency capsule (PC)—was introduced in clinical practice. Newer improved versions of the PC further eliminate obstruction risk [7–9].

## Paediatric IBD imaging studies

Multiple imaging modalities have been used in paediatric IBD, divided into conventional and cross-sectional ones.

The small bowel follow-through (SBFT) had been the most common examination of the SB, with a sensitivity and specificity of 90 and 96 % respectively for the diagnosis of CD [10]. Its use as a gold standard technique for radiological examination of the SB was justified before the introduction of cross-sectional modalities because of its high negative predictive value and ability to identify even subtle mucosal abnormalities in experienced hands.

The method with its high availability and low cost probably could still play a role in the diagnosis of IBD lesions, especially in strictures with obstruction.

SΒ enteroclysis has limited use in children because of the high radiation dose and the stress and discomfort it causes.

Both barium studies are unable to demonstrate extraluminal disease, while they have the disadvantage of a high radiation dose. Furthermore, in a number of studies, cross-sectional techniques, especially MRI, have been shown to be superior to SBFT for detecting small intestinal pathology [11, 12].

Cross-sectional techniques have advanced the ability to diagnose, classify and monitor IBD while reducing the radiation exposure. When employed under the appropriate clinical scenario, they play a crucial role: first to suggest or confirm the diagnosis of IBD in suspected cases, excluding other causes of inflammation, especially infection; second, to differentiate between IBD subtypes and contribute to the accurate classification of IBD; third, in known IBD, to evaluate response to therapy and disease activity (inflammation or/and fibrosis) and monitor progression and intestinal or extra-intestinal complications of the disease, thus suggesting the appropriate therapy. These techniques share the limitation of relatively low sensitivity to early/mild disease, restricted to the mucosa, when the predominant histological findings are erythaema and friability without ulcers because of the current limitations in spatial resolution [13].

## Ultrasound (US)

Transabdominal ultrasound (TUS) and small intestine contrast ultrasound (SICUS) are radiation-free, low-cost and easy to use radiographic techniques with high availability and good tolerance by children. They can be performed with little preparation and without sedation. Fasting 4 h before the examination is helpful. The use of oral and/or IV contrast agents remains controversial, but has been shown to be a safe and well-tolerated practice that increases diagnostic accuracy [14]. In patients with suspected CD, the sensitivity and specificity of both methods in detecting small bowel lesions are shown to be 75 and 100 % for TUS and 100 and 100 % for SICUS, while in patients with proven CD the sensitivity and specificity can reach 76 and 100 % for TUS and 96 and 100 % for SICUS respectively [15]. Another advantage of US is the real-time evaluation of bowel wall for both anatomic and functional abnormalities. Sensitivity is reported to be significantly lower for less accessible locations such as rectum (14.2 %) and duodenum/jejunum (28.6 %) [16].

**Fig. 1:**
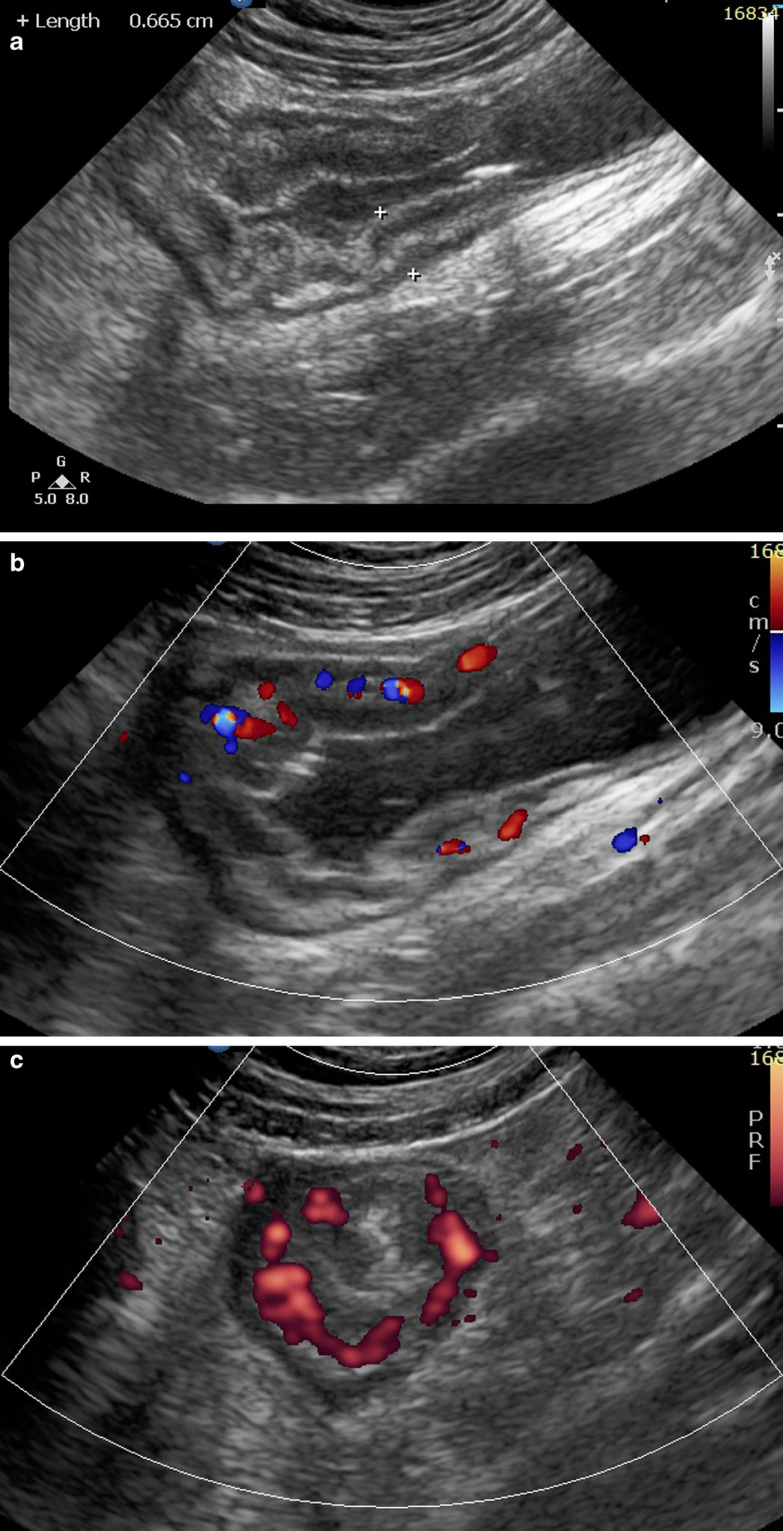

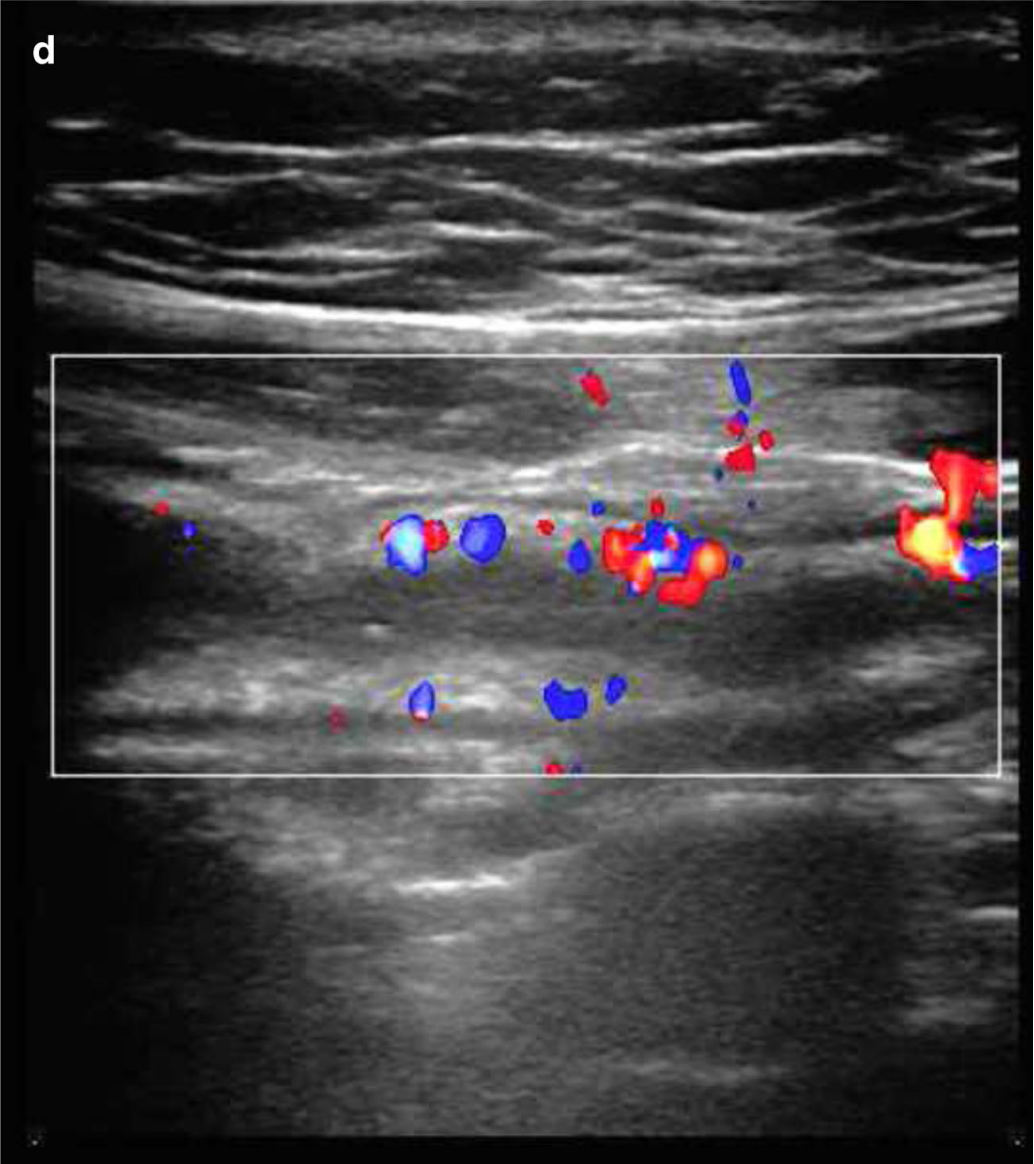
Sagittal greyscale US (**a**) in a 13-year-old female: ileocolic Crohn’s disease. Bowel wall thickening with modification of normal stratification. Sagittal colour Doppler (**b**), axial power Doppler (**c**) plane: active Crohn’s disease. Increased vascularity in the thickened bowel wall segments. Sagittal colour Doppler image (**d**) of the left colon in a 15-year-old male with UC shows thickened and moderately hyperaemic bowel wall segment with preservation of normal stratification

In general, the US protocol requires greyscale and colour Doppler imaging of the entire abdomen in the sagittal and axial planes using anterior and posterior compression techniques, at first with a high-resolution probe beginning with the terminal ileum, then examining the colon from the left to right, and finally evaluating the jejunal and ileal loops in the left upper and mid-lower abdomen respectively. Urinary bladder distension from previous oral intake enables better visualisation of intrapelvic ileal loops, while drinking water immediately before scanning enables identification of gastric and duodenal abnormalities. Finally, using a low-frequency probe, the mesentery is inspected for fluid or abscesses and solid organs for related abnormalities [17]. Ultrasound findings to look for are the following [15–20]:

- Bowel wall thickening (>3 mm) (Fig. 1a). It is one of the most important findings in IBD. Different cutoff measurements of the bowel wall have been proposed in the literature (for the terminal ileum: 1.5–3 mm; for the colon: 2–3 mm), with higher thresholds resulting in lower sensitivity and increased specificity [18]. In our department the cutoff measurement used for the SB and the colon is 3 mm.
- Modification (thickened submucosa) or loss of normal stratification.
- Bowel stiffness: non-compressible and hypoperistaltic bowel loops.
- Strictures (lumen diameter <1 cm) with prestenotic distention (lumen diameter >2.5 cm). Hyperperistalsis of the prestenotic loop is an additional finding.
- Ulcers: interruption of the submucosal hyperechoic rim by a hypoechoic tract, hyperechoic tracts perpendicular to the bowel wall or hyperechoic spots (trapped gas) in the bowel wall. Loss of stratification is also thought to be an indirect sign of chronic disease due to the development of ulcers.
- Fistulas: hypoechoic, duct-like peri-intestinal lesions with lumen diameter <2 cm.
- Abscesses: thick walled, hypoechoic, peri-intestinal round-like lesions.
- Inflammatory mesentery: appears thickened, echogenic and hyperaemic. Free fluid accumulation. Enlarged hyperemic mesenteric nodes.

Colour or power Doppler imaging of the vascularity of thickened wall segments has been proved useful in the distinction between remission and active disease, as normal bowel wall does not show much vascularity (Fig. 1b and c). The estimation of “vessel density” seems to be a reliable semiquantitative score for disease activity [21]. Additionally, patients with CD have significantly higher portal vein and mesenteric flow and a lower resistance index (RI) than controls, although these measurements have not been properly validated and are not clinically applicable [18].

Contrast-enhanced ultrasound (CEUS) is a newer diagnostic tool in paediatric IBD. Its use in the paediatric population has been reported safe [14], while in adult studies it has been shown to increase the diagnostic accuracy of detecting CD lesions and additionally to differentiate active from chronic lesions [22]. This is probably due to the unique characteristic of US contrast agents to remain inside the microcirculation and break up in the vascular system, so they are not retained in tissues that do not have an increased micro- or macrovascular network, such as the fibrous intestinal wall. More studies are needed to justify the reliability and feasibility of this promising technique in the paediatric population.

Ultrasound elasticity, although far from clinical employment in bowel wall assessment, represents a promising real-time objective diagnostic tool in the detection and measurement of fibrosis in IBD. So far, it has been shown that it can accurately differentiate inflammatory from fibrotic bowel in rat models of IBD [23].

The clinical role of US in UC is less well established compared with CD. Mural stratification is preserved in most UC patients because of the mucosal/submucosal pattern of inflammation. Bowel wall thickening is also a characteristic feature (Fig. 1d). Pathologic RI measurements in the inferior mesenteric artery related to disease activity have been reported [19].

Amongst the disadvantages of US are the facts that the examination is operator dependent and not reproducible and that it is difficult to examine the whole GI tract, with additional difficulties in obese children and in case of gas fullness of the bowel.

### Key points

Being radiation free, low cost and easy to use and having high availability, good tolerance, and high sensitivity and specificity for terminal ileum lesions [20] make ultrasound a first-line imaging technique for IBD especially for (1) screening, (2) suspected IBD, particularly when MRE is not possible because of young age or need for sedation, and (3) evaluation of post-treatment changes, particularly in cases of isolated terminal ileum disease.

## Computed tomography (CT)

CT examination by either CT enterography (CTE) or CT enteroclysis has become a widely used technique in adults for SB investigation. The latter is rarely performed especially in children because it is more invasive and probably of similar diagnostic accuracy, as shown in an adult study [24].

CTE has become the preferred imaging technique for evaluating IBD because of certain advantages over MRI: shorter examination time, convenient procedure, greater availability, increased radiologist familiarity and experience in interpreting findings, high spatial resolution, fewer motion artefacts, less need for sedation, lower cost and availability for patients with implanted MR-sensitive devices [25].

Despite the well-established benefits of CTE, there is increasing concern about the radiation risk and consequent malignancy risk, especially in this already predisposed paediatric population. Therefore, mainly for radiation protection reasons, MDCT is avoided by most paediatric radiologists. However, iterative reconstruction algorithms in CTE have shown that a decrease of effective doses to less than 2 mSv is possible with considerably lower image quality but without missing clinically significant diagnostic information [26, 27]. The feasibility and integration into daily clinical practice of these low-dose techniques require further investigation and standardisation.

**Fig. 2:**
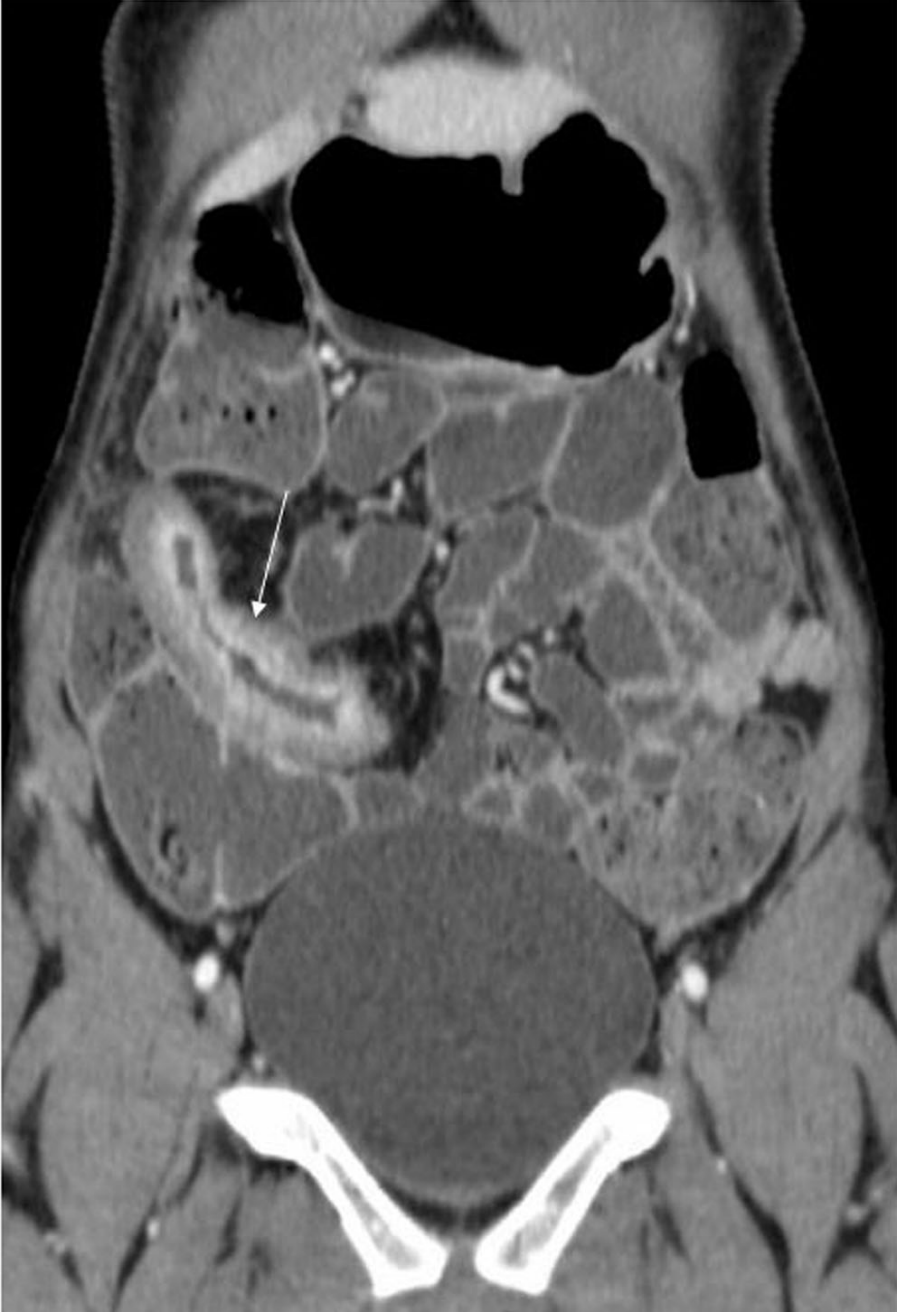
Crohn’s disease: coronal CTE. Terminal ileum involvement in a 14-year-old male: bowel wall thickening, submucosal oedema, lumen stenosis and increased mucosal enhancement

**Fig. 3:**
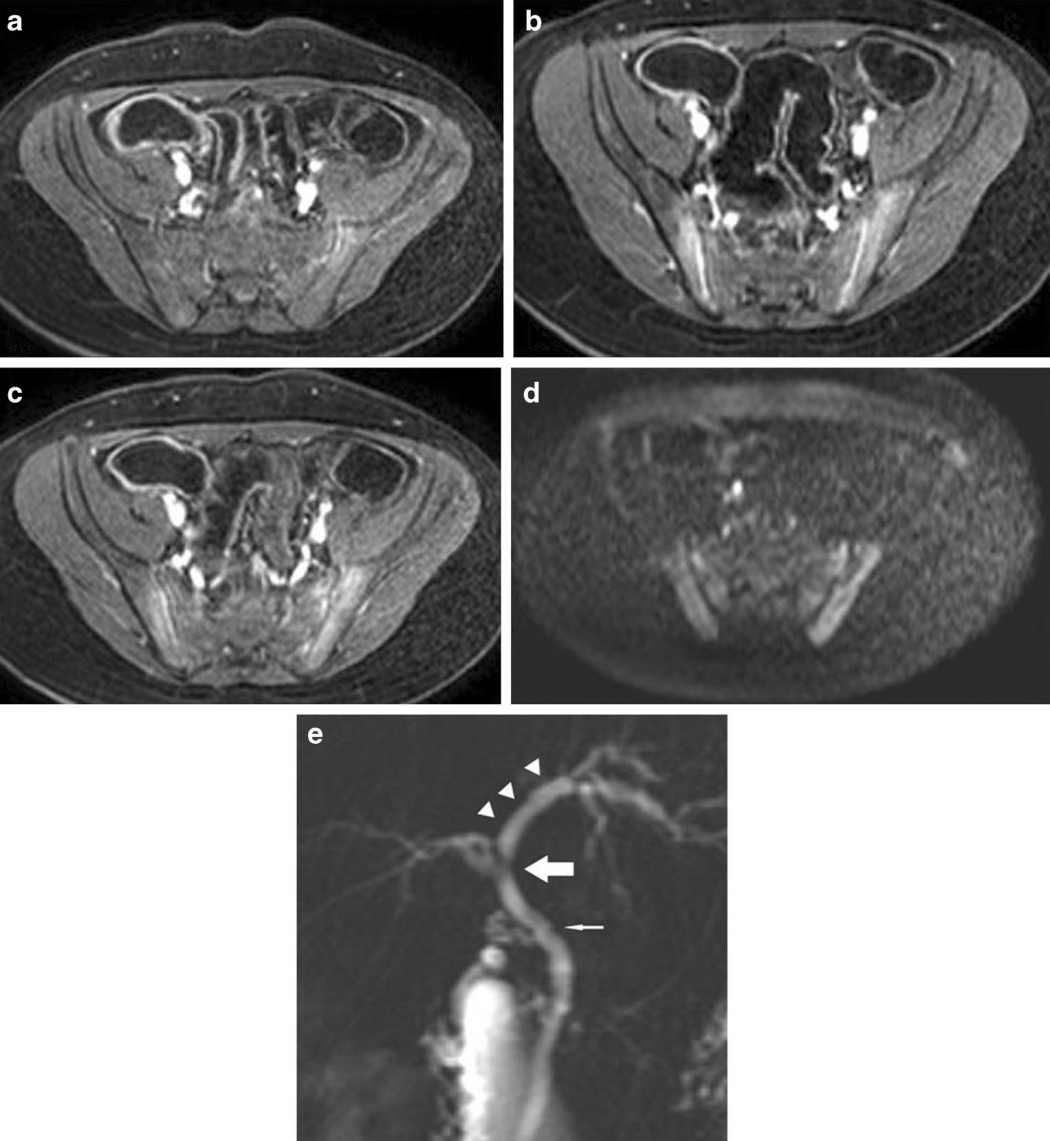
Extraintestinal manifestations of Crohn’s disease in a 14-year-old male. Sacroilitis in Crohn’s disease. Increased contrast enhancement (**a, b, c**) and restricted diffusion (**d**) in the sacroiliac joints. Focal irregular stenosis in the common bile duct (*thin arrow*) and hepatic bile duct confluence (*thick arrow*). Pre-stenotic dilatation of the left hepatic duct (*arrowhead*). The imaging findings are typical of primary sclerosing cholangitis in CD (**e**)

CT findings to look for include [13, 21, 24, 28]:

- Bowel wall thickening greater than 3 mm in a distended bowel loop.
- Mural hyperenhancement (Fig. 2) and segmental hyperenhancement of the small bowel wall compared with the adjacent small bowel loops. Using a mural attenuation threshold of 109 HU and an abnormal to normal loop enhancement ratio of more than 1.3, CTE is highly correlated with histological findings of active disease. Visual assessment, however, presents higher specificity than quantitative measurements do [21].
- Mural stratification due to intramural oedema is more indicative of active disease compared to a homogeneously enhanced wall, while the presence of submucosal fat indicates a more chronic process.
- The “comb sign”. Increased attenuation of the mesenteric fat is due to oedema and engorgement of the vasa recta.
- Chronic fibrostenosing disease. Strictures without mural hyperenhancement or other signs of active inflammation.
- Sacculations. The inflammatory process of CD usually affects the mesenteric border of the bowel loops, so when fibrosis is established, stricturing and shortening of the mesenteric side result in compensatory dilatation of the anti-mesenteric wall.
- Fibrofatty proliferation.
- Sinus tracts or fistulas. Fistulas with oedematous origins that are not visible on SBFT may be detected at CT as linear enhancing tracts with or without communication with adjacent structures, tethering bowel loops. The most common type of fistula in CD is a perianal fistula. Other fistula types include enterocutaneous, rectovaginal, enterovaginal and enterovesicular. Lifetime risk of fistulous disease in CD is 20–40 % [29].
- Abscesses are easily depicted on CTE.
- Treatment response. Mural hyperenhancement and bowel wall thickening, the most sensitive signs of active disease, are significantly decreased after therapy. The pattern of mural stratification usually changes from stratification with mucosal hyperenhancement toward homogeneous enhancement.
- CT has low sensitivity for detection of ulcers. Deep mural ulcers may present as focal bowel wall defects that contain fluid or oral contrast material. Adjacent bowel wall hyperenhancement is usually seen.
- Inflammatory pseudopolyps can be seen in distended bowel.
- Pneumatosis, unsuspected perforation and mural thinning in patients with toxic megacolon.
- Extraenteric manifestations of CD can also be evaluated with CT or MR imaging (Fig. 3): sclerosing cholangitis, cholelithiasis, liver abscess, portal vein thrombosis, pancreatitis, hydronephrosis caused by ureteral involvement, nephrolithiasis, IBD-related arthropathy (progressive ankylosing spondylitis and sacroiliitis), osteoporosis, peritoneal pseudocysts and cutaneous manifestations [30].

### Key points

CTE, due to its radiation risk, is mainly preferred for acute emergency situations, assessing potential complications of IBD that require surgical management, such as perforation, peritonitis, post-operative leaks, abscess, severe strictures/obstruction and fistulas. It remains an examination of choice for abscess drainage, used in order to obtain more accurate images of the exact extension and select the most appropriate access route.

## Magnetic resonance imaging (MRI)

MR enterography (MRE) of the small bowel is a recent technique that is currently widely applied in the adult population and to a significant degree in children. It is performed either as MR enterography or MR enteroclysis. In children, because of its simplicity and the lack of radiation exposure (no need to advance a catheter in the jejunum under fluoroscopic guidance), MR enterography is the preferred technique. However, problems such as imaging artefacts due to bowel peristalsis and motion or poor cooperation of the child with the oral contrast agent can cause an examination to be of poor quality. For these reasons the technique is preferably applied in patients above the age of 9, while there is no need for sedation [31]. According to a recent meta-analysis in the paediatric population, the sensitivity and specificity of MR enterography in active CD has been reported to be 84 and 97 % respectively [32, 33].

**Fig. 4:**
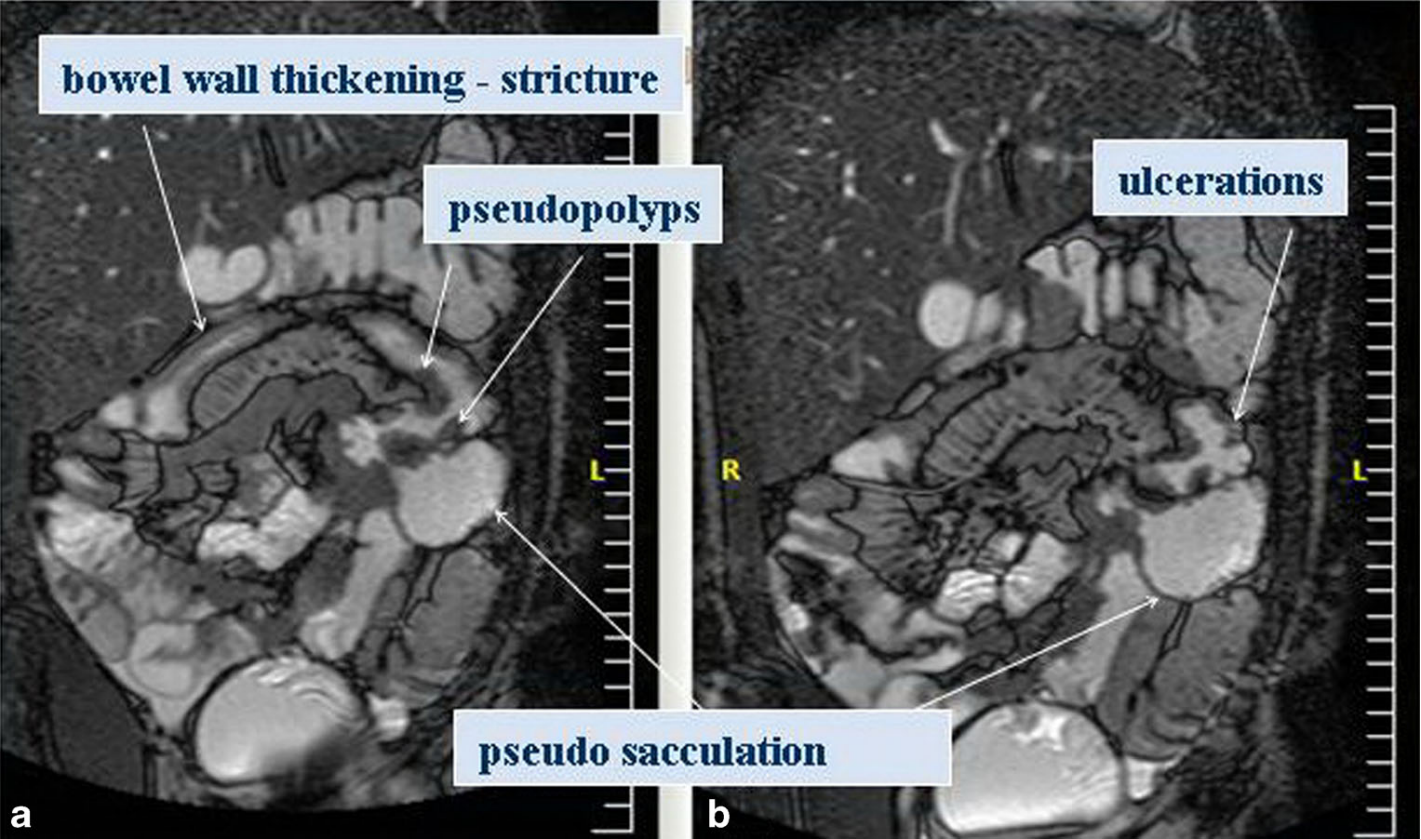
Coronal T2 steady-state acquisition image in a 14-year-old male. Bowel wall thickening, strictures, ulceration, pseudopolyps and pseudosacculation

There are still controversies concerning the prediction of disease activity because of the lack of a gold standard examination for comparison, the different MR techniques used and the fact that acute and chronic lesions may coexist in the same bowel loop (Fig. 4). However, MRI has been shown to be the best imaging technique to differentiate active inflammation from fibrosis. For active disease, the sensitivity and specificity of MRE are estimated as 87.5 and 79.3 % compared to 100 and 62.1 % for CTE, while for fibrosis, the sensitivity and specificity of MRE are 57.1 and 82.1 % compared to 42.3 and 67.9 % of CTE respectively [28].

**Fig. 5:**
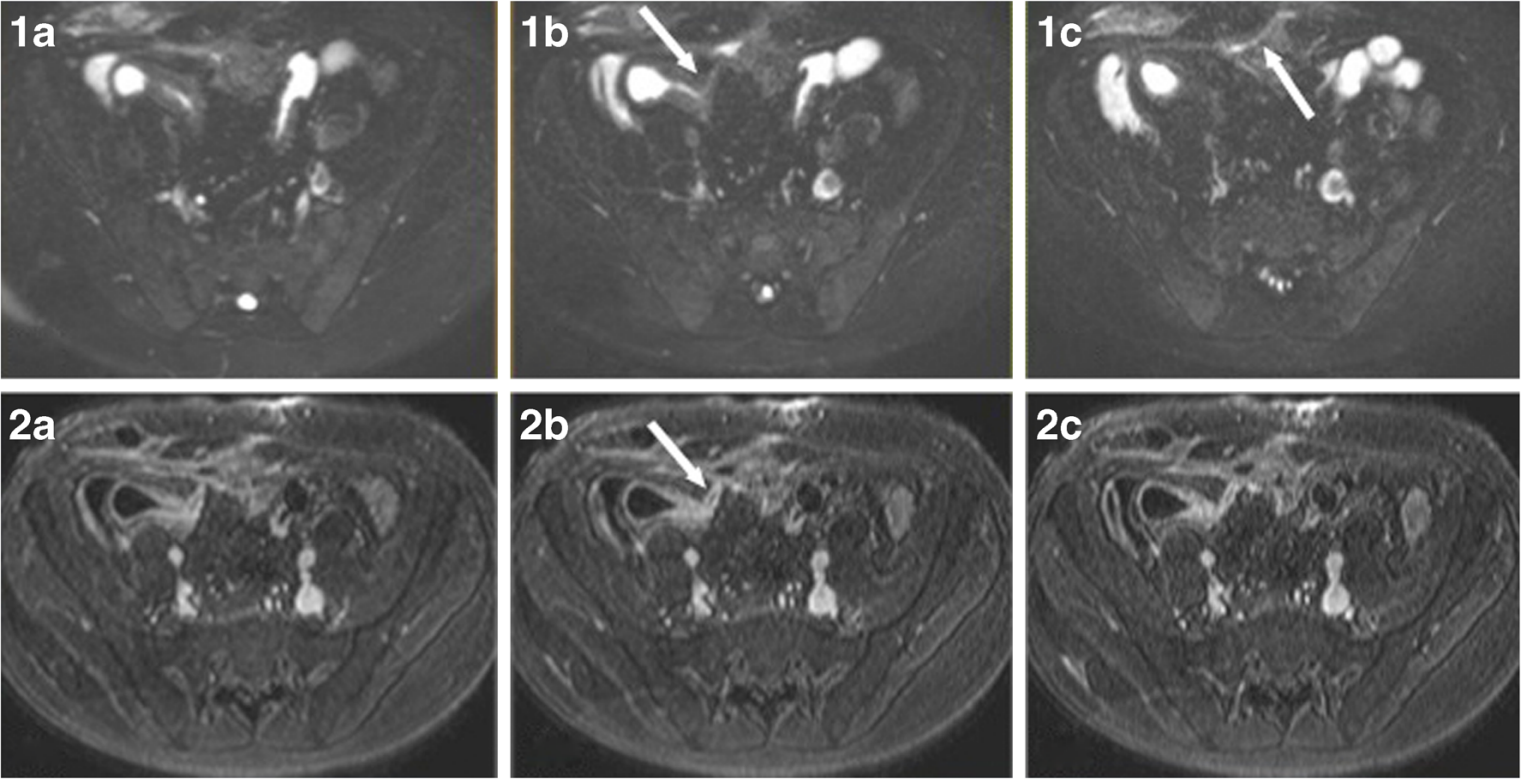
Ileocutaneous fistula in a 13-year-old male. T2 fat-saturated (1**a–c**) and T1 contrast-enhanced (2**a–c**) images. Direct visualisation of the fistula (*arrows*) is feasible as it shows avid contrast enhancement and contents of increased T2 signal, suggestive of enteric material

**Fig. 6:**
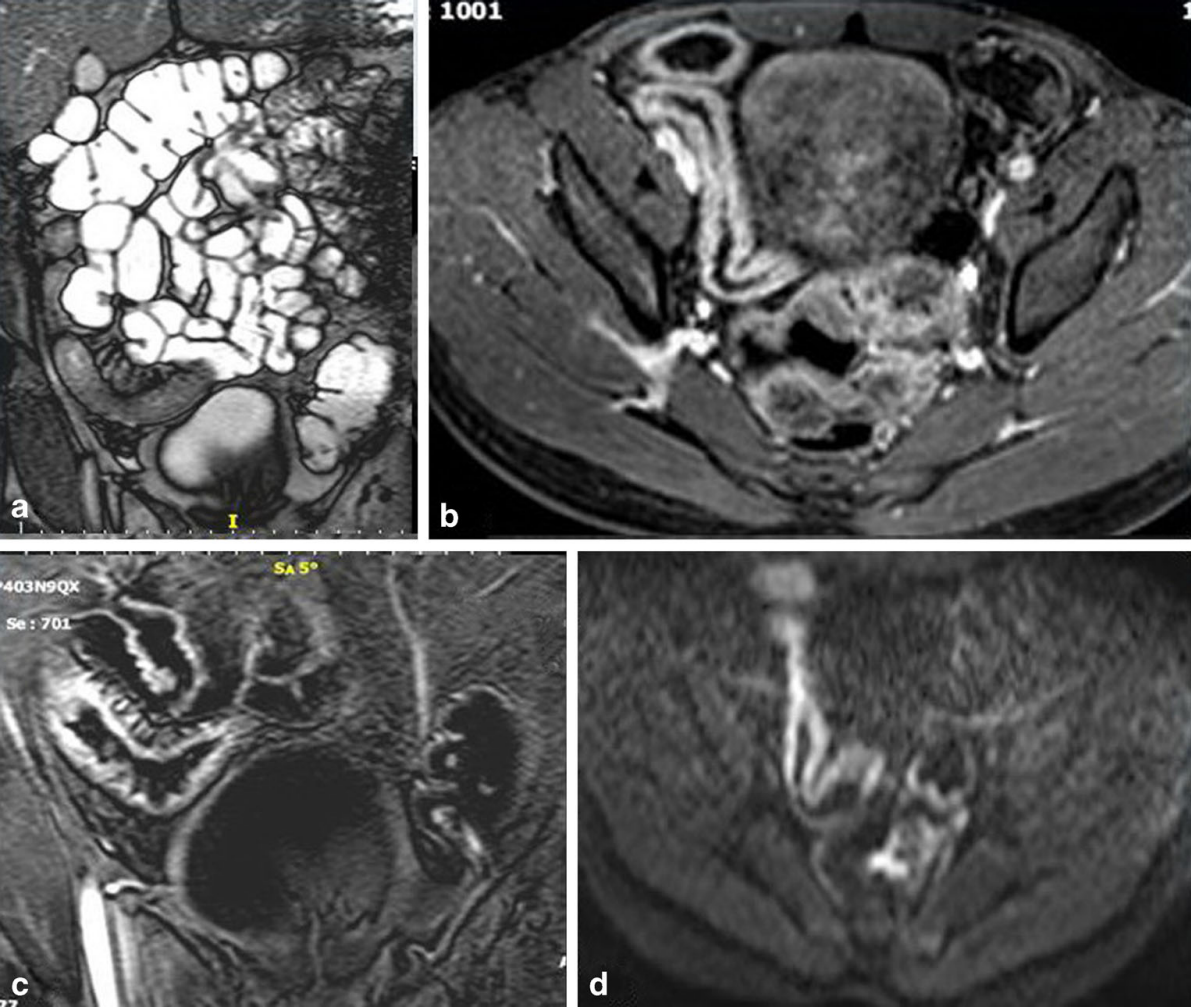
Crohn’s disease: terminal ileum involvement in a 14-year-old female. Disease activity. Increased signal of the bowel wall in coronal T2 steady-state acquisition (**a**) with restricted diffusion (**d**). Prominent early mucosal enhancement on post-gadolinium T1W images in 30 s (**c**) and more homogeneous enhancement in 120 s (**b**)

Specific MR findings suggestive of acute active inflammatory stage in CD include [28, 29] (Figs. 5 and 6):

- Bowel wall thickening (greater than 3 mm) with increased signal in the abnormal bowel wall and adjacent mesentery on T2W fat-suppressed images.
- Early intense mucosal enhancement followed by progressive transmural enhancement on post-gadolinium T1W images. Layered wall morphology has been attributed to exacerbation of mucosal inflammation in chronic disease, while homogeneous enhancement is more common in newly diagnosed CD [34].
- Ulcers and fistulas are best seen on fast imaging with steady-state precession. They show avid contrast enhancement (Fig. 5).
- Reactively enlarged (>5 mm in short axis) adjacent mesenteric nodes often exhibit contrast enhancement and high signal on diffusion-weighted image (DWI).
- DWI sequences with high b-values open new horizons in the detection and quantification of bowel wall inflammation. In the adult population, detection of IBD with DWI sequences has a sensitivity of 95 % and specificity of 82 %, which is the highest sensitivity but lowest specificity ever reported compared with previous techniques. Neubauer et al. showed that DWI in combination with T2W is at least equal to CE-MRI for detecting acute lesions in CD (Fig 6). Based on these two sequences, imaging without the need of contrast media seems to be sufficient for diagnosis, reducing the scanning time to less than 10 min [34]. Promising attempts have been made to measure CD activity quantificatively using DWI and CE-MRI.

**Fig. 7:**
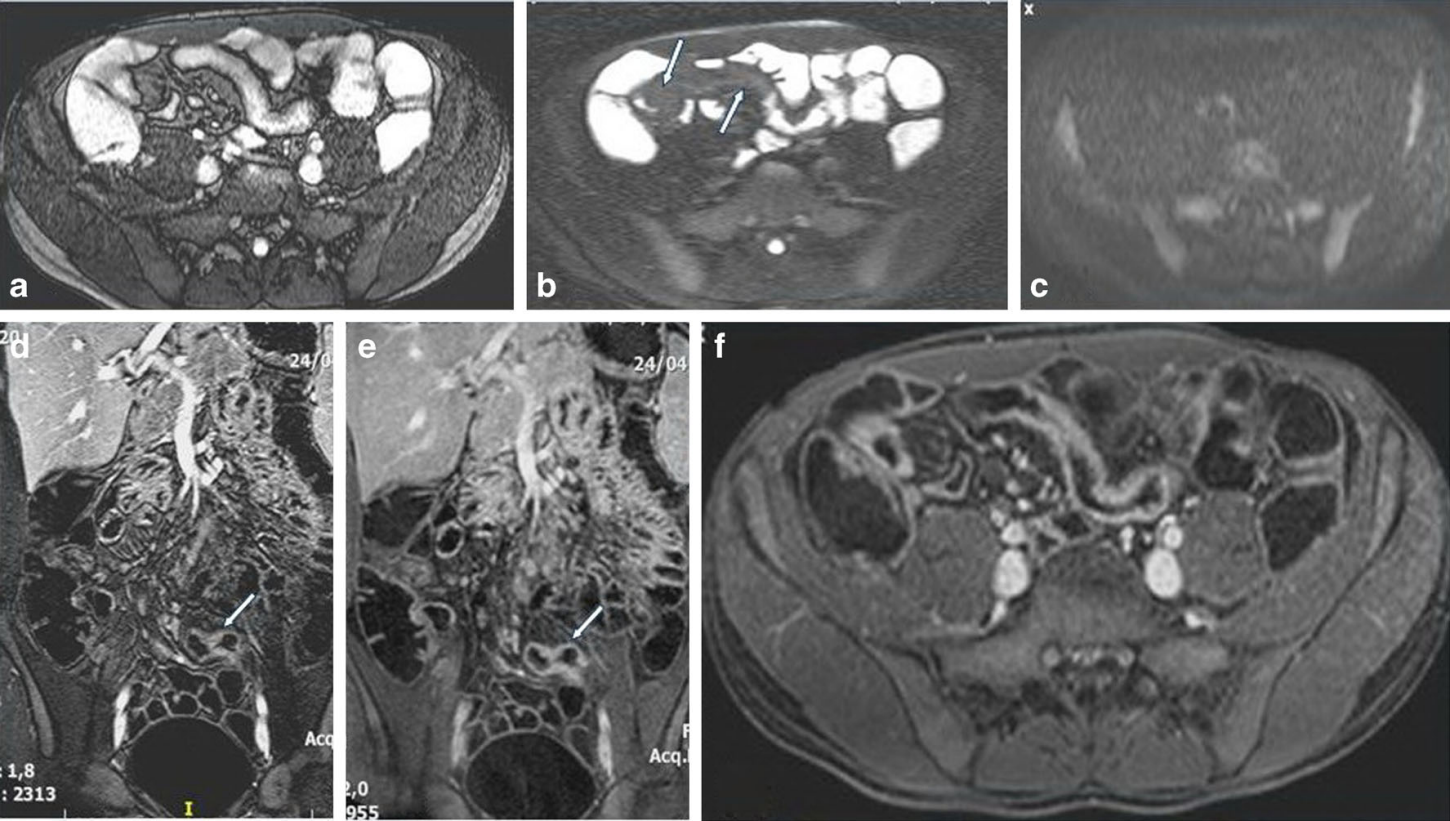
Disease chronicity in a 16-year-old male. Low signal intensity of the bowel wall in the axial T2-steady state acquisition image (**a**) and fat-saturated T2 image (**b**) without restricted diffusion (**c**). Delayed progressive homogeneous enhancement on coronal delayed arterial (**d**), coronal (**e**) and axial (**f**) equilibrum phase images

Chronic disease without active inflammation (Fig. 7).

- Bowel wall thickening and homogeneous slow enhancement on delayed post gadolinium T1W images with low signal intensity on T2W FS images. Strictures and obstruction are usually evident.
- New sequences have been applied to specifically detect fibrosis. The magnetisation transfer (MT) technique reflects data of a different set of molecular properties than standard T1 and T2 imaging. MT MRI is a quantificative measurement of the energy transferred from protons in free mobile water molecules compared to protons in water molecules associated with large molecules such as proteins and collagen. Therefore, fibrotic tissue exhibits a high MT effect, which—as shown by Adler et al. in animal studies—correlates with the degree of fibrosis and the amount of type I collagen. Pazahr et al. showed that MT can be measured in the human bowel wall with promising results using a 2D encoded gradient-echo sequence with a MT prepulse. The short acquisition time makes it attractive for everyday clinical practice [35, 36].

Motility imaging is a new MRI technique, quantificating a functional parameter, with high correlation to histopathology and shown to be more accurate than conventional MRI in the detection and severity grading of lesions in both active and chronic CD. Although more studies are required to establish the feasibility of this technique in clinical practice and for classification of motility disorders, it seems a promising tool for assessing the severity of disease [37].

Extraintestinal manifestations of IBD are easily detectable with MRI; sclerosing cholangitis, one of the most serious ones, with a relatively high prevalence in IBD patients (~4.5 %), has been shown to be easily and accurately investigated with MR cholangiopancreatography (MRCP). MRCP is a non-interventional method that also has the benefit of no need for sedation or exposure to radiation and comparable results to the gold standard technique, ERCP [30, 38].

### Key points

MRI is a radiation free technique with diagnostic accuracy at least comparable to that of CT. Although limited clinical data exist, it has been shown that MRI is the most sensitive technique to differentiate fibrosis from active inflammation. Recent technical advances in body MRI, including the 3-T magnetic field, parallel image processing and motion artefact reduction techniques, raise hopes for shorter scan times and increased spatial resolution for detecting early inflammatory changes [28, 39].

## Conclusion

US should be the first choice examination in children with suspected IBD and should be performed before endoscopy, while MR enterography is the technique of choice in children with known IBD for the investigation of the small bowel and the whole GI tract, as it is a reproducible and well-tolerated examination, lacking radiation and providing excellent information about bowel disease. CT should be reserved for cases where MRI is contraindicated, for non-cooperative younger children or in acute-emergency situations when US is inadequate. SBFT currently has a questionable role as it seems to be widely replaced by MR enterography in the paediatric population, despite its lower cost. It should be used for very young children where performing either MR or CT enterography is impossible.
